# Adrenarche-accompanied rise of adrenal sex steroid precursors prevents NAFLD in Young Female rats by converting into active androgens and inactivating hepatic Srebf1 signaling

**DOI:** 10.1186/s12864-024-10107-6

**Published:** 2024-02-19

**Authors:** Haoqing Li, Yingyu Liu, Fengyan Meng, Junan Chen, Xingfa Han

**Affiliations:** 1 College of Life Science, Sichuan Agricultural University, Ya’an, 625014 China; 2 College of Animal Science and Technology, Sichuan Agricultural University, Chengdu, 611130 China

**Keywords:** NAFLD, Adrenarche, De novo lipogenesis, Srebf1, Rat

## Abstract

**Background:**

Non-alcoholic fatty liver disease (NAFLD) has rapidly become the most common cause of chronic liver disease in children and adolescents, but its etiology remains largely unknown. Adrenarche is a critical phase for hormonal changes, and any disturbance during this period has been linked to metabolic disorders, including obesity and dyslipidemia. However, whether there is a causal linkage between adrenarche disturbance and the increasing prevalence of NAFLD in children remains unclear.

**Results:**

Using the young female rat as a model, we found that the liver undergoes a transient slowdown period of growth along with the rise of adrenal-derived sex steroid precursors during adrenarche. Specifically blocking androgen actions across adrenarche phase using androgen receptor antagonist flutamide largely increased liver weight by 47.97% and caused marked fat deposition in liver, thus leading to severe NAFLD in young female rats. Conversely, further administrating nonaromatic dihydrotestosterone (DHT) into young female rats across adrenarche phase could effectively reduce liver fat deposition. But, administration of the aromatase inhibitor, formestane across adrenarche had minimal effects on hepatic de novo fatty acid synthesis and liver fat deposition, suggesting adrenal-derived sex steroid precursors exert their anti-NAFLD effects in young females by converting into active androgens rather than into active estrogens. Mechanistically, transcriptomic profiling and integrated data analysis revealed that active androgens converted from the adrenal sex steroid precursors prevent NAFLD in young females primarily by inactivating hepatic sterol regulatory element-binding transcription factor 1 (Srebf1) signaling.

**Conclusions:**

We firstly evidenced that adrenarche-accompanied rise of sex steroid precursors plays a predominant role in preventing the incidence of NAFLD in young females by converting into active androgens and inactivating hepatic Srebf1 signaling. Our novel finding provides new insights into the etiology of NAFLD and is crucial in developing effective prevention and management strategies for NAFLD in children.

**Supplementary Information:**

The online version contains supplementary material available at 10.1186/s12864-024-10107-6.

## Background

Non-alcoholic fatty liver disease (NAFLD) is increasingly recognized as a leading cause of liver damage and cirrhosis in developed countries [1]. It is the hepatic manifestation of the metabolic syndrome characterized by excessive accumulation of fat in the liver of individuals who consume little or no alcohol [2]. NAFLD is commonly associated with type 2 diabetes mellitus [3], cardiovascular disease [4] and chronic kidney disease [5], and can progress to more severe liver disease including steatohepatitis (NASH), hepatic cirrhosis/fibrosis and even hepatocellular carcinoma [6]. Now, NAFLD has rapidly become the most common cause of chronic liver disease in children and adolescents [2], affecting up to 10% of the pediatric population worldwide [6]. Unfortunately, there are currently no effective clinical measures or strategies available for the prevention and management of NAFLD/NASH, which seriously affects the physical and mental health of children [7]. Thus, NAFLD/NASH in children and adolescents has become a major challenge for modern society [2, 8]. Although several factors including genetic, epigenetic, environmental and lifestyle factors have been reported to contribute towards the incidence and progression of NAFLD in children [2, 9], its pathogenesis is complex and still largely unclear. To effectively prevent and manage NAFLD in children and adolescents, it is crucial to have a comprehensive understanding of its etiology.

Adrenarche is a developmental event that occurs during childhood and marks the onset of steroid hormone production in the adrenal glands, and it has been evidenced to be a critical stage for hormonal changes [10]. Thus, any disruptions during this period have been linked to metabolic abnormalities and diseases, such as obesity and dyslipidemia [10–12]. However, whether there is a causal linkage between adrenarche disorders and the increasing prevalence of NAFLD in children remains unknown. Here, we firstly reported that adrenarche-accompanied rise of sex steroid precursors plays a key role in preventing the incidence of NAFLD in early life of females by converting into active androgens and inactivating hepatic sterol regulatory element-binding transcription factors 1 (SREBF1, also known as SREBP1) signaling. This novel finding provides new insights into the etiology of NAFLD and is crucial in developing effective prevention and management strategies for NAFLD in children.

## Results

### Liver undergoes a transient slowdown period of growth during adrenarche in young female rats

**Fig. 1:**
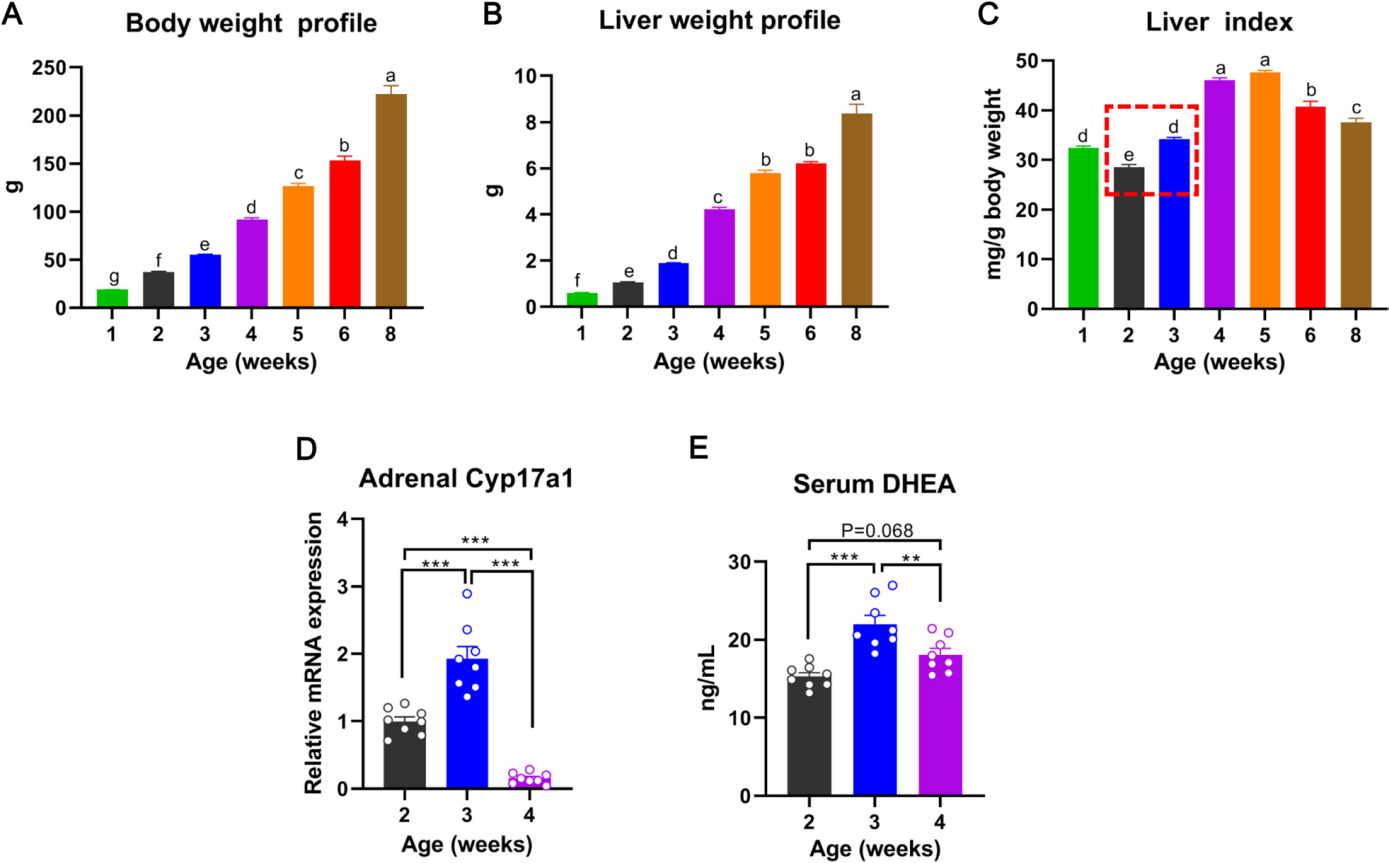
The liver undergoes a transient slowdown period of growth during adrenarche in young female rats. **A** Body growth profile of female rats from neonatal to adult age. **B** Liver growth profile of female rats from neonatal to adult age. **C** Liver index profile of female rats from neonatal to adult age. Red dotted rectangle indicates the short transient slowdown period of liver growth in young female rats. **C** Adrenal cytochrome P450 17A1 (*Cyp17a1*) mRNA expression profile in female rats during 2 to 4 wk of age. **D** Serum dehydroepiandrosterone (DHEA) concentration profile in female rats during 2 to 4 wk of age. ^a−g^ Means without a common letter differed (*p* < 0.05), ** *p* < 0.01, *** *p* < 0.001

“Hepatostat theory” highlights that the liver-to-body-weight ratio needs to be maintained always at 100% of what is required for body homeostasis [13]. Disruption of the normal liver-to-body-weight ratio greatly raises the risk of developing metabolic syndromes. To profile liver growth and development, we measured the body and liver weight weekly in female rats from neonatal to adult age. In consistent with the “hepatostat theory”, the liver weight increased proportionally with the body weight from neonatal to adult age in female rats (Fig. 1A and B). Unexpectedly, the liver-to-body-weight ratio (i.e., liver index) [14] of female rats undergoes a transient slowdown period of growth during 2–3 wk of age (Fig. 1C).

Previous studies reported that the period around postnatal 16–20 days is the adrenarche phase in young female rats [15]. To confirm whether the short transient slowdown period of liver growth is during adrenarche in young female rats, we quantified the mRNA expression of adrenal cytochrome P450 17A1 (*Cyp17a1*), the determinant factor for adrenarche onset [15], and serum dehydroepiandrosterone (DHEA) concentrations during 2–4 wk of age in female rats. Resultantly, the mRNA expression of *Cyp17a1* in adrenal glands markedly increased and peaked (*p* < 0.001) at 3 wk of age, then quickly dropped (*p* < 0.001) to almost undetectable levels at 4 wk of age (Fig. 1D). In parallel, serum concentrations of DHEA markedly increased and peaked (*p* < 0.001) at 3 wk of age, and then dropped (*p* < 0.01) to low levels at 4 wk of age (Fig. 1E). Therefore, liver growth undergoes a transient slowdown period of growth during adrenarche in young female rats.

### Blocking androgen actions during adrenarche promotes liver growth in young female rats

**Fig. 2:**
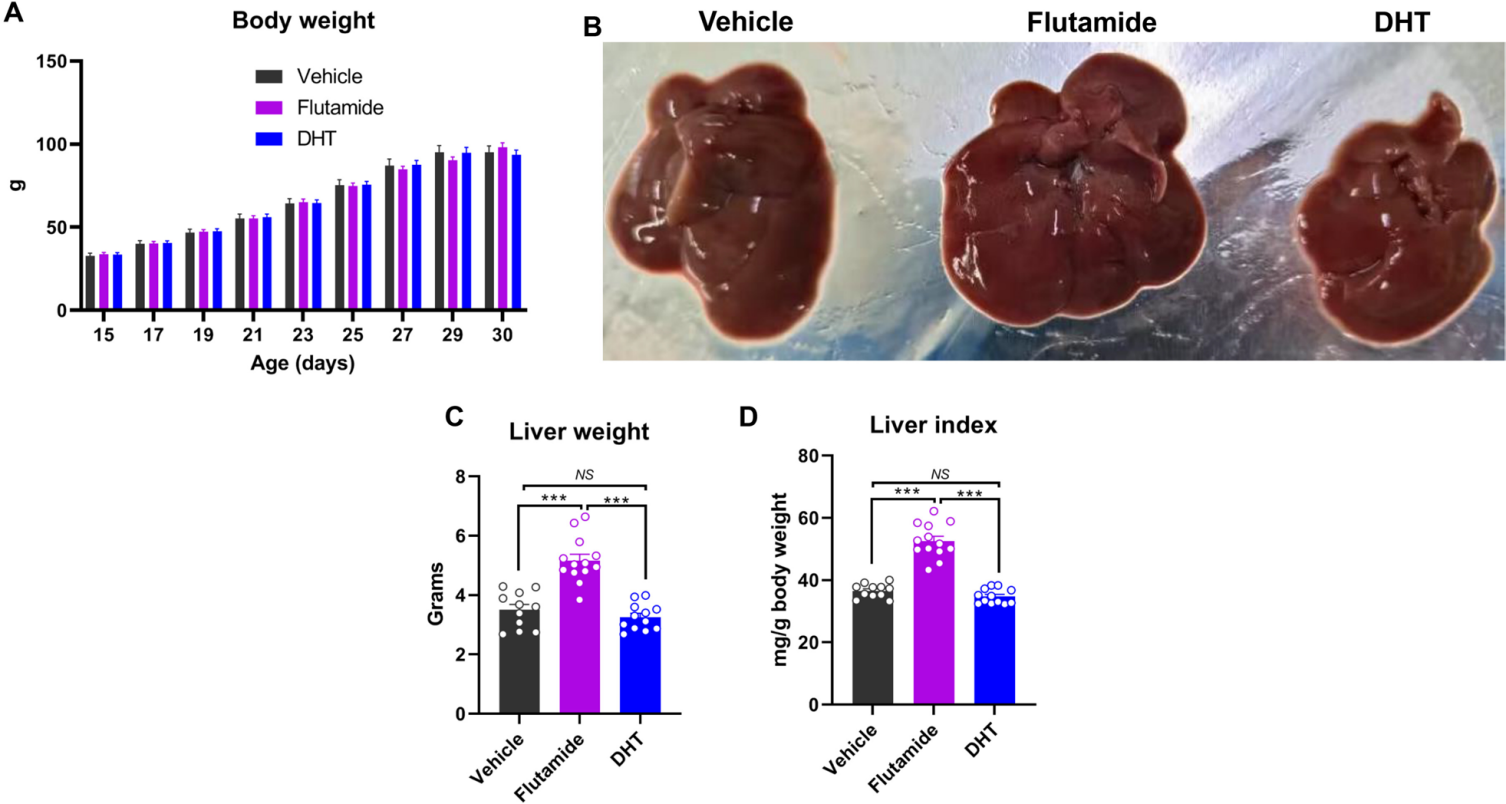
Adrenarche-accompanied rise of androgens prevents liver growth during adrenarche in young female rats. **A** Both flutamide (androgen receptor antagonist) and dihydrotestosterone (DHT) administration had minimal effects on body growth during adrenarche in young female rats. B Representative images of the liver of young female rats following flutamide or DHT administration. **C**-**D** Blocking androgen actions by flutamide administration across adrenarche largely increased liver weight and liver index in young female rats. *NS*, not significant, *** *p* < 0.001

During adrenarche, the adrenal glands secrete increased levels of sex steroid precursors including DHEA, dehydroepiandrosterone sulfate (DHEA-S), and androstenedione (A4), but without increased corticosterone levels [15]. The most abundant sex steroid precursor produced during adrenarche is DHEA [16], which is biologically inactive [17] and only converted into active sex steroid hormones (i.e., active androgens and/or active estrogens) in the specific target tissues, e.g., liver where the appropriate enzymatic machinery exists [14]. To confirm whether DHEA or other adrenal-derived sex steroid precursors could convert into active androgens and then prevent liver growth during adrenarche in young female rats, we administrated female rats with flutamide (androgen receptor antagonist) at a dose of 50 mg/kg [18] every two days from 14- to 21-day of age, which covered the whole adrenarche phase in female rats. Compared to vehicle-treated controls, flutamide administration had no effects on body growth (*p* > 0.05; Fig. 2A), but markedly increased (*p* < 0.001) both liver weight and liver index (Fig. 2B-D). Remarkably, the average liver weight and liver index were increased by 47.97% and 43.66%, respectively by flutamide administration in relative to vehicle-treated controls. And, further dihydrotestosterone (DHT) administration across adrenarche phase numerically reduced both liver weight and index in young female rats (Fig. 2B-D). These results strongly evidenced that active androgens converted from the adrenal-derived sex steroid precursors play a predominant role in preventing liver growth during adrenarche in young female rats.

### Transcriptomic profiling verified that the liver growth during adrenarche in young female rats is very susceptible to androgens

**Fig. 3:**
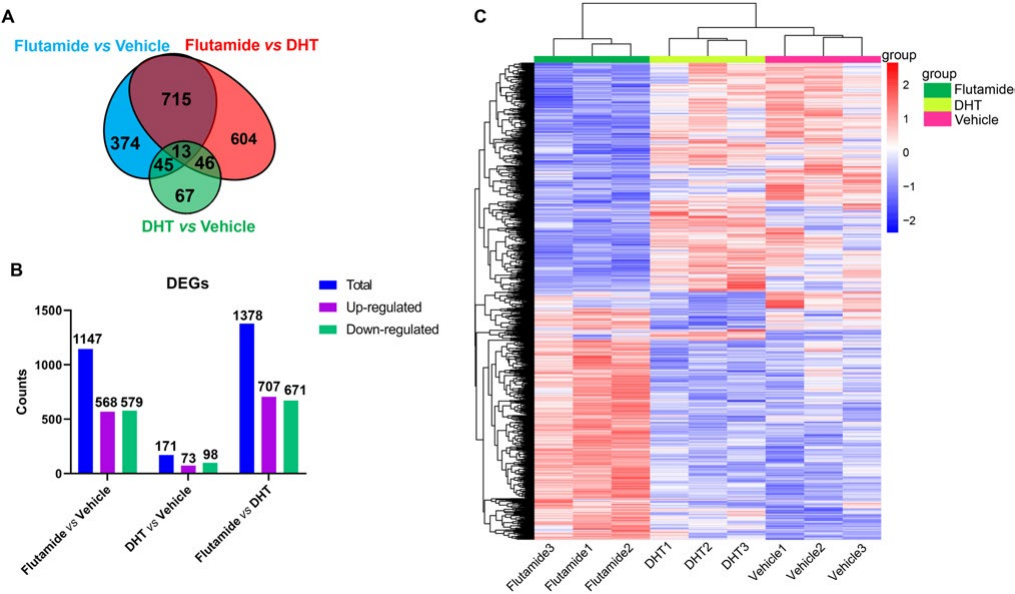
Transcriptomic profiling verified that the liver growth in young female rats during adrenarche is very susceptible to androgens. **A** Venn diagram of differentially expressed genes (DEGs) among groups. **B** The number of DEGs between pairwise groups. **C** Hierarchical clustering analysis of the differentially expressed genes (DEGs) based on the z-score of their FPKM value

To gain insights into the causal linkage between adrenarche and liver growth retardation in young female rats, we conducted comparative transcriptomic investigations of the livers of young female rats flowing flutamide, DHT or vehicle administration during adrenarche. Pairwise comparison among groups identified totally 1864 differentially expressed genes (DEGs, Fig. 3A; see Methods for criteria). Of those, 1147 DEGs were identified between flutamide- versus vehicle-treated rats, of which 568 (49.52%) were upregulated and 579 (50.48%) were downregulated in flutamide-treated rats (Fig. 3B; Supplemental file 1). A few more (i.e., 1378) DEGs were identified between flutamide- versus DHT-treated females, of which 707 (51.31%) were upregulated and 671 (48.69%) were downregulated in flutamide-treated rats. And, only 171 DEGs were identified between DHT- versus vehicle-treated female rats, of which 73 (42.69%) were upregulated and 98 (57.31%) were downregulated in DHT-treated females (Fig. 3B; Supplemental file 1).

Hierarchical clustering of those DEGs showed that the gene expression patterns between flutamide-treated females and vehicle-treated controls were almost completely opposite (Fig. 3C), revealing that active androgens converted from adrenal-derived sex steroid precursors during adrenarche play great roles in orchestrating liver’s physiology and function. Further enhancing androgen actions across adrenarche in young female rats by administrating DHT still caused a clear separation in gene expression patterns of livers from vehicle-treated controls (Fig. 3C), also reinforcing the liver’s physiology and metabolism in female rats during adrenarche are very susceptible to active androgens.

### Adrenarche-accompanied rise of androgens causes liver growth retardation in young female rats by inhibiting hepatic de novo fatty acid biosynthesis and blocking cell cycle progression

To further gain insights into the mechanism linking adrenarche-accompanied rise of androgens with liver growth retardation, functional enrichment analysis of these hepatic DEGs regulated by flutamide/DHT administration was conducted using DAVID (v2023q1).

**Fig. 4:**
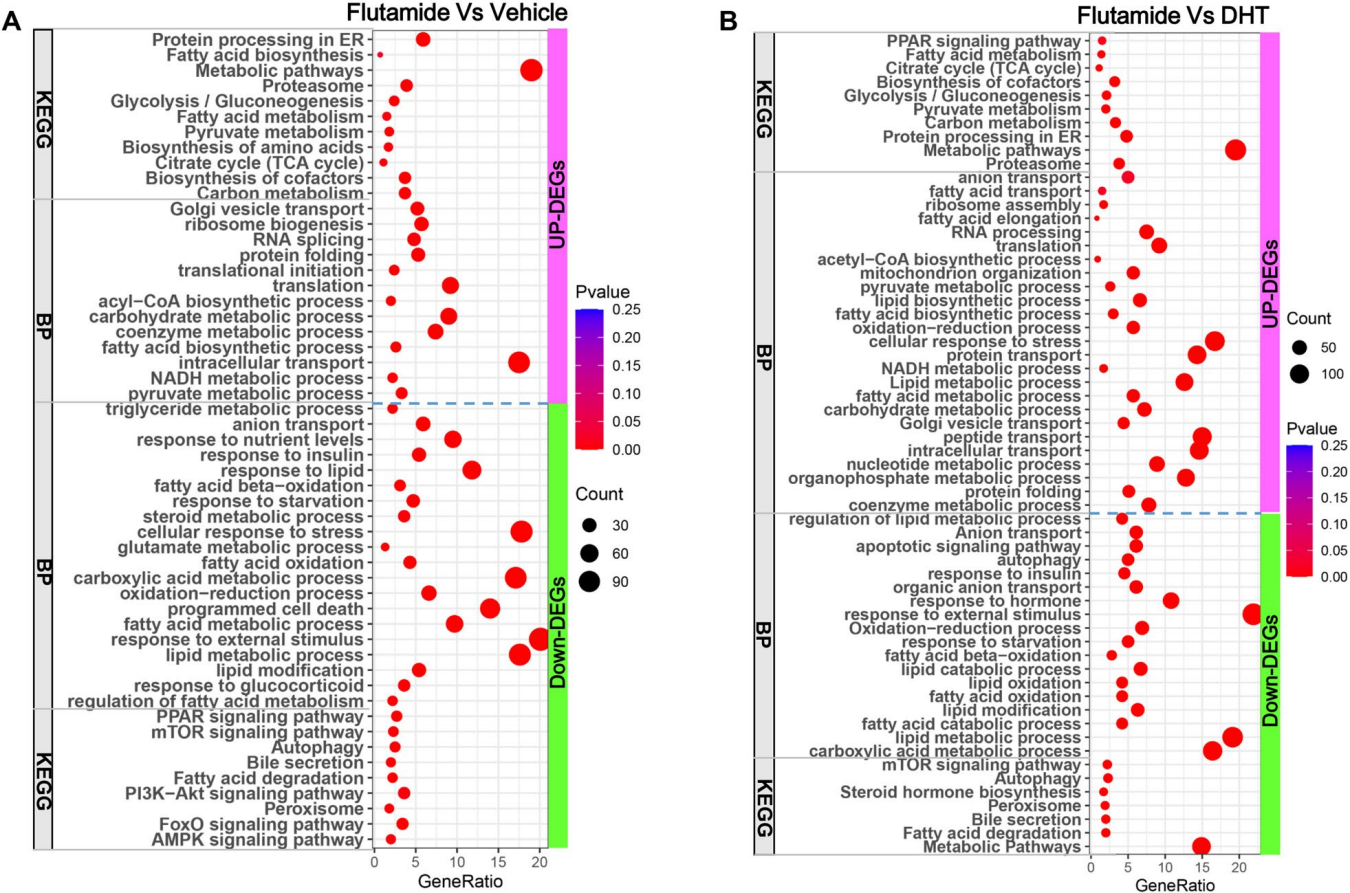
Functional enrichment analysis of DEGs indicate that blocking androgen actions during adrenarche promoted de novo lipogenesis and meanwhile suppressing fatty acid oxidation in liver of young female rats. **A** Functional enrichment analysis of the DEGs between flutamide- versus vehicle-treated rats using DAVID (v2022q4). **B** Functional enrichment analysis of the DEGs between flutamide- versus DHT-treated rats using DAVID (v2022q4)

Comparing females treated with flutamide versus vehicle, the upregulated DEGs in liver were mainly annotated into biological process (BP) of carbohydrate metabolic process, pyruvate metabolic process, acetyl-CoA biosynthetic process, fatty acid biosynthetic process, NADH metabolic process, ribosome biogenesis, RNA splicing, translation, protein folding, nitrogen compound transport, etc., into molecular function (MF) of ribonucleoside binding, anion binding, RNA binding, nucleoside phosphate binding, etc., into cellular component (CC) of mitochondrion, endoplasmic reticulum, pyruvate dehydrogenase complex, transport vesicle, etc., and into KEGG of metabolic pathways, glycolysis, pyruvate metabolism, fatty acid biosynthesis, TCA cycle and protein processing in ER (Fig. 4A; Supplemental file 2). While, the down-regulated DEGs were mainly annotated into BP of lipid metabolic process, fatty acid oxidation, carboxylic acid metabolic process, response to insulin/glucocorticoid, anion transport, programmed cell death, response to external stimulus, etc., into MF of coenzyme binding, anion binding, cofactor binding, etc., into CC of mitochondrion, endoplasmic reticulum, cell junction, lysosome, vacuole, etc., and into KEGG of metabolic pathways, AMPK signaling pathway, FoxO signaling pathway, fatty acid degradation, autophagy, bile secretion and mTOR signaling pathway (Fig. 4A; Supplemental file 2).

Functional enrichment analysis of the DEGs between flutamide- versus DHT-treated females also indicated that flutamide administration enhanced de novo fatty acid biosynthesis and decreased fatty acid oxidation in liver as well (Fig. 4B; Supplemental file 2). Besides, gene set enrichment analysis (GSEA) using all identified genes between groups also verified that blocking androgen actions promoted ATP citrate lyase (ACLY)-mediated Ac-CoA biosynthesis and suppressed fatty acid beta-oxidation in livers (Supplemental file 3). These results strongly suggested that blocking androgen actions during adrenarche using flutamide potently enhanced liver de novo fatty acid synthesis and simultaneously suppressed liver fatty acid oxidation in young female rats.

**Fig. 5:**
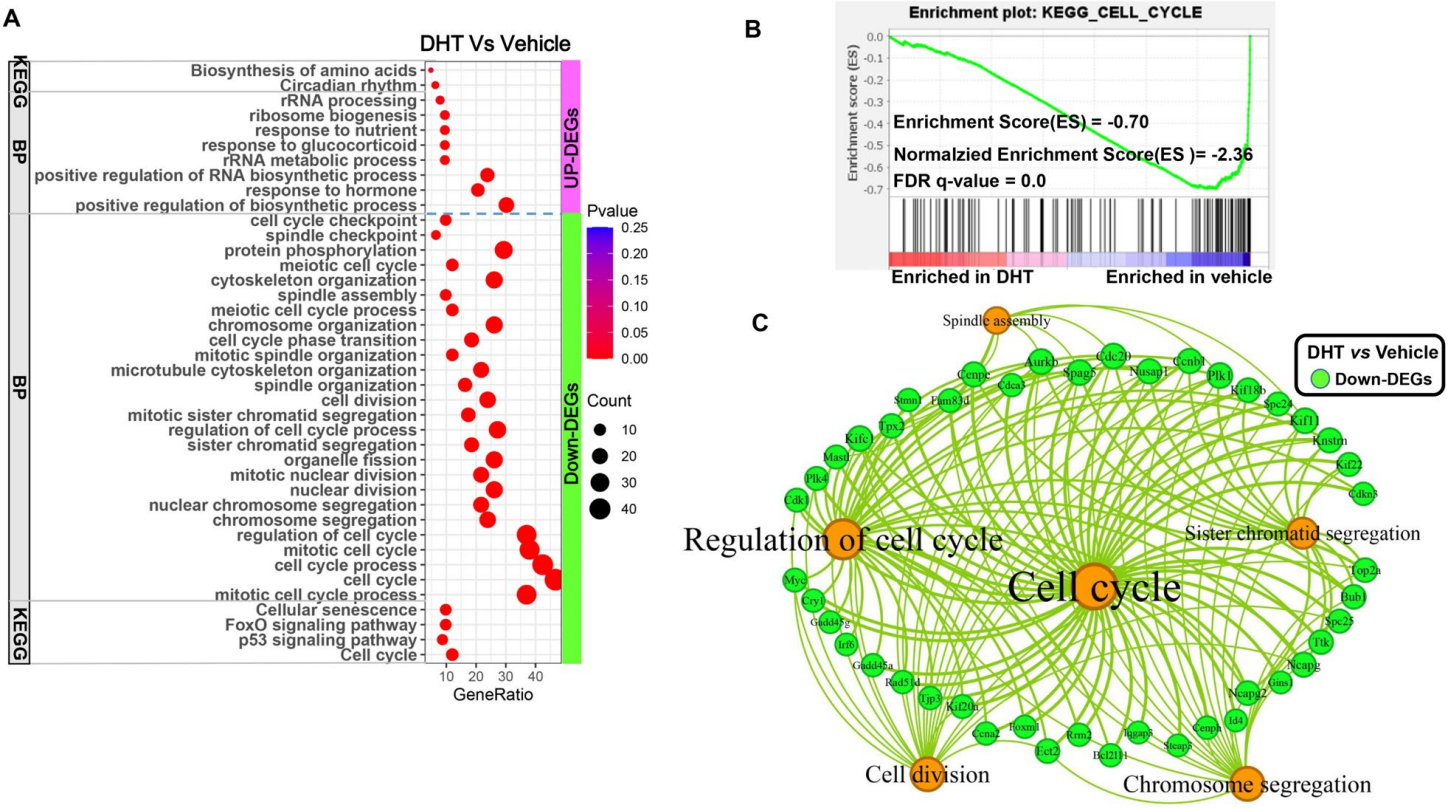
Enhancing androgen actions by DHT administration across adrenarche blocked hepatic cell cycle in young female rats. **A** Functional enrichment analysis of the DEGs between DHT- versus vehicle-treated rats using DAVID (v2022q4). **B** Gene set enrichment analysis reinforced that hepatic cell cycle was suppressed by DHT administration during adrenarche in young female rats. **C** Network analysis identified key terms and key DEGs involved in cell cycle regulated by DHT administration

Comparing DHT-treated females versus vehicle-treated controls, the up-regulated DEGs were mainly annotated into BP of ribosome biogenesis, rRNA processing, and into KEGG of biosynthesis of amino acids (Fig. 5A). While the down-regulated DEGs were predominantly annotated into BP of cell cycle, regulation of cell cycle, chromosome segregation, organelle fission, cell division, cell cycle phase transition, spindle assembly, etc., into CC of spindle, chromosome, microtube cytoskeleton, kinetochore, centrosome, etc., into MF of microtube binding, tubulin binding, cytoskeletal protein binding, ATP binding, etc., into KEGG of cell cycle, FoxO signaling pathway, cellular senescence, etc. (Fig. 5A; Supplemental file 2). GSEA analysis using the whole identified genes between DHT- and vehicle-treated females also reinforced that DHT administration significantly suppressed hepatic cell cycle in young female rats (Fig. 5B). These results strongly indicated that DHT administration across adrenarche predominantly suppressed hepatic cell cycle progression in young female rats. A large quantity of down-regulated DEGs (e.g., *Kifc1*, *Kif11*, *Kif18b* and *Kif22*) which were annotated into cell cycle, regulation of cell cycle, cell division, chromosome segregation and/or spindle assembly, were identified and shown in Fig. 5C. Therefore, adrenarche-accompanied rise of androgens causes liver growth retardation predominantly by inhibiting hepatic de novo fatty acid synthesis as well as cell cycle progression.

### Blocking conversion of adrenal sex steroid precursors into active androgens during adrenarche caused severe NAFLD in young female rats

**Fig. 6:**
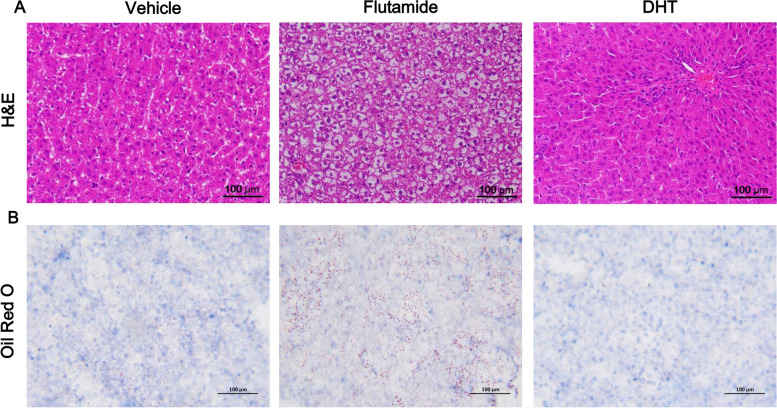
Flutamide administration across adrenarche caused severe NAFLD in young female rats. **A** Representative images of the liver section stained with HE (×20 magnification). **B** Representative images of the liver section stained with Oil Red O (×20 magnification)

Given functional enrichment analysis indicated that blockage of androgen actions during adrenarche using flutamide predominantly promoted de novo fatty acid biosynthesis in liver of young female rats, we then performed histological analysis to check fat deposition in liver. Surprisingly, both H&E (Fig. 6A) and Oil Red O staining (Fig. 6B) evidently indicated that flutamide administration across adrenarche phase resulted in severe fat deposition in liver, causing significant non-alcoholic fatty liver disease (NAFLD) in young female rats. In contrast, further DHT administration across adrenarche decreased fat deposition in liver in young female rats (Fig. 6B).

**Fig. 7:**
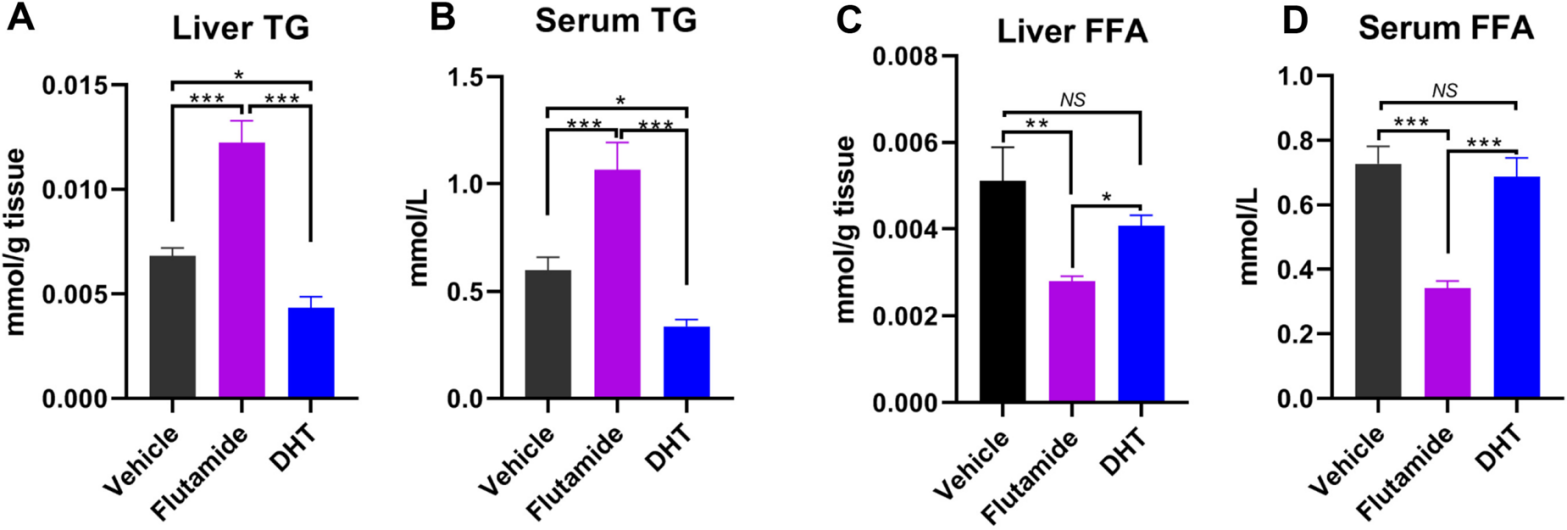
Flutamide administration across adrenarche in young female rats largely increased fat deposition in liver. **A** Liver triglyceride (TG) content. **B** Serum TG concentrations. **C** Liver free fatty acid (FFA) content. **D** Serum FFA concentrations. NS, not significant, * *p* < 0.05, ** *p* < 0.01, *** *p* < 0.001

Quantification assays further verified that flutamide administration during adrenarche predominantly increased (*p* < 0.001) liver triglyceride (TG) content, while DHT administration decreased (*p* < 0.05) liver TG content (Fig. 7A). In parallel, serum TG concentrations were increased (*p* < 0.001) in young female rats following flutamide administration but decreased (*p* < 0.05) following DHT administration (Fig. 7B). In contrast, free fatty acid (FFA) in both liver and serum was markedly decreased (*p* < 0.001) by flutamide administration (Fig. 7C), but not affected by DHT administration in young female rats (*p* > 0.05; Fig. 7C and D). These findings strongly suggest that the adrenarche phase is a crucial period susceptible for developing NAFLD in young females. And, the rise of adrenal-derived androgens during this phase appears to be very critical in preventing the incidence of NAFLD in early life of females.

### Blocking the conversion of adrenal sex steroid precursors into active estrogens across adrenarche exerts minimal effects on liver lipogenesis and lipid deposition in young female rats

**Fig. 8:**
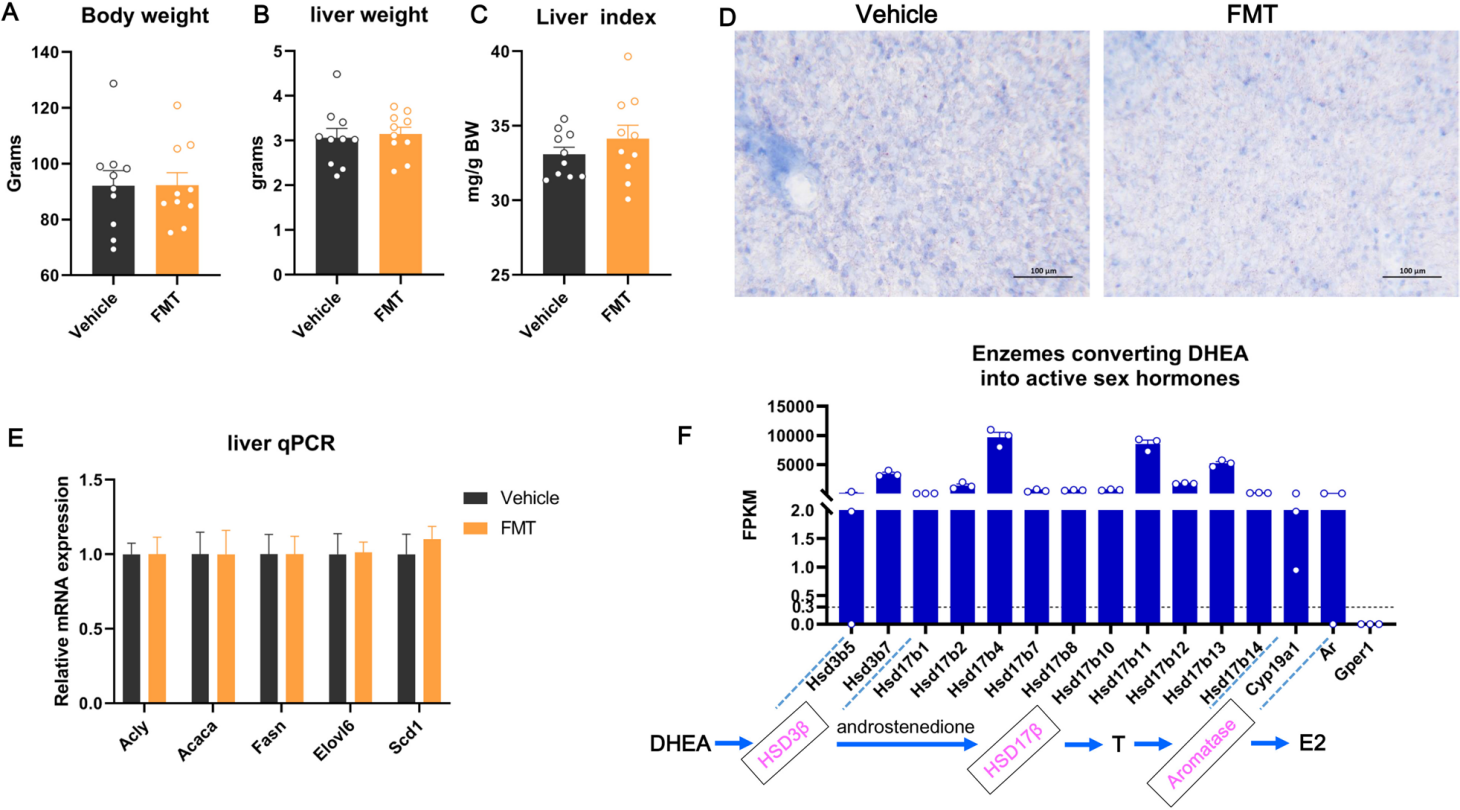
Formestane administration during adrenarche had minimal effects on liver lipogenesis and lipid deposition in young female rats. Formestane (FMT) administration across adrenarche exerted minimal effects on body weight **A**, liver weight (**B**) and liver index (**C**) in young female rats. (**D**) Representative images of the liver section stained with Oil Red O (×20 magnification). (E) mRNA expression of key lipogenic genes in liver of young female rats administrated with formestane (FMT) or vehicle. **F** Expression of key genes mediating the conversion of DHEA into both active androgens and estrogens as well as sex hormone receptor encoding genes in liver of young female rats

Previous studies have evidenced that the most abundant adrenal-derived sex steroid precursor DHEA could exert great effects on preventing NAFLD by converting into active estrogens rather than active androgens within the liver organ in adult female mice [14]. To check whether adrenarche-accompanied rise of sex steroid precursors like DHEA could prevent NAFLD by converting into active estrogens in liver of young female rats, we administrated young female rats with aromatase inhibitor, formestane from 2 to 4 wk of age, which totally covered the whole adrenarche phase. As a result, formestane administration across adrenarche phase had minimal effects on liver growth (Fig. 8A-C) and liver lipid deposition as evidenced by Oil red O staining (Fig. 8D), suggesting that adrenarche-accompanied rise of adrenal sex steroid precursors could not prevent NAFLD by converting into active estrogens in young females. To further validate this outcome, we performed qPCR to check the expression of key lipogenic genes in liver. Consistently, the mRNA expression of all determined key lipogenic genes (e.g., *Acly*, *Acaca*, *Fasn*, *Elovl6* and *Scd1*) in liver of young female rats was not affected by formestane administration (Fig. 8E).

Adrenal-produced sex steroid precursors like DHEA can rapidly metabolize into androstenedione under the catalysis of 3β-hydroxysteroid dehydrogenase (3β-HSD) in liver. Then, androstenedione can be converted into testosterone (T) by the 17β-hydroxysteroid dehydrogenase (17β-HSD), and testosterone could be subsequently converted into 17β-estradiol (E2) by the aromatase (encoded by the *Cyp19a1* gene) in liver [14]. To further understand the underlying mechanisms why adrenarche-accompanied rise of adrenal sex steroid precursors could not prevent NAFLD by converting into active estrogens, we checked the expression of key genes mediating the conversion of adrenal sex steroid precursors (e.g., DHEA) into active androgens/estrogens in liver in our transcriptome data. Consequently, using the critical criteria (FPKM ≥ 0.3) for gene expression, all key enzymes mediating the conversion of DHEA into either active androgen, i.e., testosterone (T) or active estrogen i.e., 17β-estradiol (E2) were all highly expressed in liver of young female rats (Fig. 8F; Supplemental file 1). Therefore, adrenal-derived sex steroid precursors like DHEA could be converted into both active androgen and estrogen in liver of young female rats. Then, we checked the expression of receptors for both active androgens and estrogens. Previous studies have confirmed that the active estrogens converted from adrenal DHEA prevent NAFLD only by precisely activating G protein-coupled estrogen receptor (GPER1, also called GRP30) but not ESR1 or ESR2 within the liver organ [14]. Surprisingly, we found that the young female rat liver did not express *Gper1* but *Ar* (Fig. 8F; Supplemental file 1). Therefore, deficiency of expression of *Gper1* in liver could well explain why adrenal sex steroid precursors could not prevent NAFLD in young female rats by converting into active estrogens. Taken together, all these results suggest that adrenarche-accompanied rise of adrenal sex steroid precursors exert great effects on preventing NAFLD by converting into active androgens rather than active estrogens in young female rats.

### Overlapping analysis identifies key genes in mediating adrenarche-accompanied androgens to prevent NAFLD in young female rats during adrenarche

**Fig. 9:**
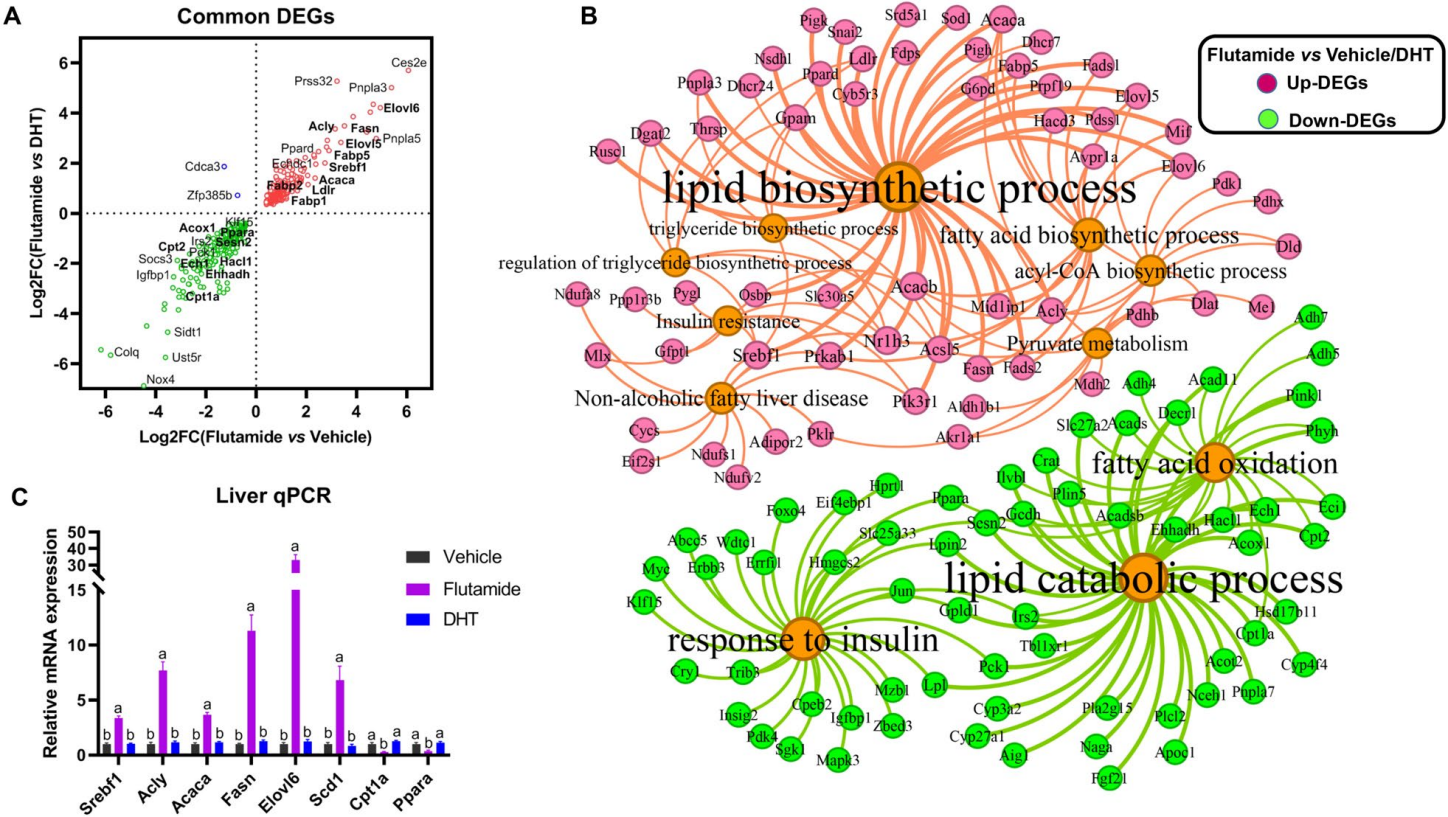
Overlapping analysis identifies key genes in mediating adrenarche-accompanied androgens to prevent NAFLD in young female rats during adrenarche. **A** The common regulated DEGs by flutamide in comparison to vehicle or DHT. **B** Network analysis identified key terms and key DEGs involved in liver fat metabolism regulated by active androgens. **C** qPCR validation of the selected key DEGs involved in fatty acid biosynthesis/oxidation. Different letters indicate *p* < 0.05. *Srebf*1, sterol regulatory element-binding tran-scription factor 1; *Acly*, ATP citrate lyase; *Acaca*, acetyl-CoA carboxylase alpha; *Fasn*, fatty acid synthase; *Elovl6*, ELOVL fatty acid elongase 6; *Scd1*, stearoyl-Coenzyme A desaturase 1; *Cpt1a*, carnitine palmitoyltransferase 1 A; *Ppara*, peroxisome proliferator activated receptor alpha

To identify key genes that mediate adrenal-derived androgens to prevent NAFLD in young female rats during adrenarche, we performed overlapping analysis to identify the common DEGs between flutamide- versus vehicle-treated rats, and between flutamide- versus DHT-treated rats. Totally, 728 common DEGs were identified, of which 382 DEGs were commonly upregulated, 344 DEGs were commonly downregulated, and the remaining 2 DEGs (i.e., *Cdca3* and *Zfp385b*) were conversely regulated (Fig. 9A). A cluster of well-described de novo fatty acid biosynthesis genes (e.g., *Srebf1*, *Acl*y, *Fasn*, *Acaca*, *Elovl6* and *Scd1*) were detected among the common upregulated DEGs (Fig. 9A). While, a series of well-described fatty acid oxidation genes (e.g., *Cpt1a*, *Ppara*, *Acox1* and *Ech1*) were identified among the common downregulated DEGs. Functional enrichment analysis indicated that these common upregulated DEGs were predominantly enriched into de novo fatty acid & lipid synthesis-associated terms, e.g., pyruvate metabolism, acyl-CoA biosynthesis, fatty acid biosynthetic process, triglyceride biosynthesis process, etc., and into KEGG of non-alcoholic fatty liver disease and insulin resistance (Fig. 9B). In contrast, the the common downregulated DEGs were predominantly enriched into BP of lipid catabolic process and fatty acid oxidation, and into KEGG of response to insulin (Fig. 9B).

To further validate our findings, we selected a group of key genes involved in de novo fatty acid biosynthesis/fatty acid oxidation to verify their expression by qPCR. In consistent with the RNA-seq results, these selected key genes associated with de novo fatty acid biosynthesis in liver were all predominantly upregulated, whereas these selected key genes associated with fatty acid oxidation were all downregulated by flutamide administration (Fig. 9C). While, all of these selected genes were minimally affected by DHT administration (Fig. 9C).

### Integrative data analysis reveals that Srebf1 plays a central role in mediating adrenarche-accompanied androgens to prevent NAFLD in young females

Active androgens trigger cellular responses through binding to androgen receptor (AR), which is a nuclear transcription factor [19]. Using CiiiDER software [20], we searched for DEGs with potential binding sites for AR. Promoter regions spanning 1500 bp upstream and 500 bp downstream of the transcription start site were identified. Resultantly, only two DEGs, i.e., *Ppara* and *Ehhadh* involved in fatty acid oxidation were with putative AR binding sites. Surprisingly, no DEGs involved in either lipid biosynthetic process or cell cycle were identified to contain putative AR binding sites, revealing that most of androgens’ effect on preventing liver de novo lipogenesis and cell cycle is attained by indirect signaling pathways.

**Fig. 10:**
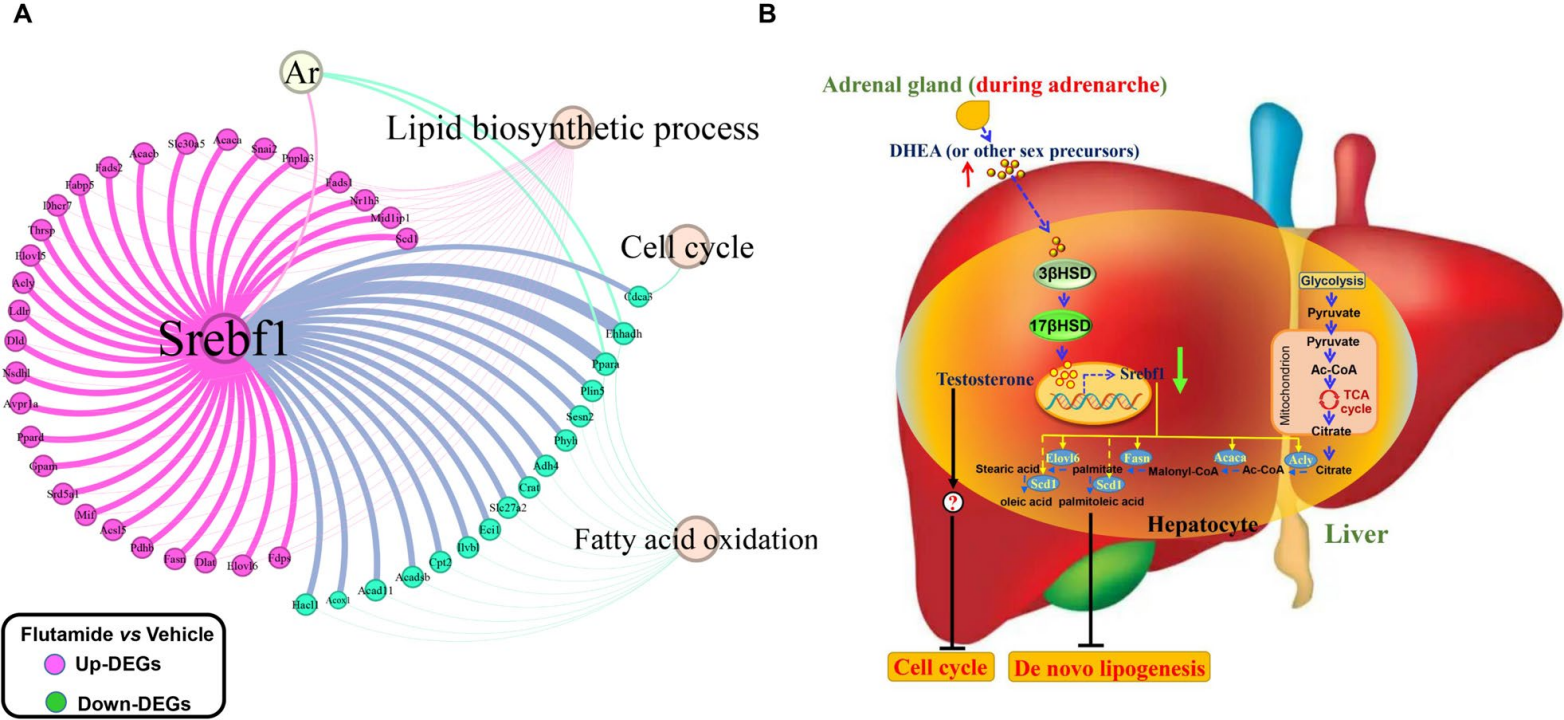
Integrated data analysis identified that Srebf1 plays a central role in mediating adrenarche-accompanied androgens to prevent NAFLD in young females. **A** Most of the key DEGs involved in lipid biosynthetic process/fatty acid oxidation are with putative SREBF1 binding sites within their promoter regions. Promoter regions spanning 1500 bp upstream and 500 bp downstream of the transcription start site were scanned using CiiiDER software. **B** The putative mechanism by which adrenarche-accompanied androgens in pre-venting NAFLD in young females

Previous published articles have evidenced that *Srebf1* is the AR transcriptional target gene [21] and simultaneously it is a well-known transcription factor to promote *de no* lipogenesis in liver [22]. We then searched for DEGs with potential binding sites for SREBF1 with CiiiDER using the same protocols described above. As a result, many well-known DEGs involved in de novo fatty acid biosynthesis (e.g., *Acly*, *Acaca*, *Fasn*, *Elovl6*, *Scd1*) [22] as well as DEGs involved in fatty acid oxidation (e.g., *Ppara*) were with putative SREBF1 binding sites (Fig. 10A). However, only one DEG, i.e., *Cdca3* involved in cell cycle was identified to be with putative SREBF1 binding sites (Fig. 10A).

Previous study indicated that adrenal androgen precursors like DHEA can be converted into androstenedione by 3β-HSD, and then to testosterone by 17β-HSD [23], and the expression of both the two enzyme encoding genes were determined in liver by RNA-seq (Fig. 8F). Given hepatic glycolysis, pyruvate metabolism, acyl-CoA biosynthesis and TCA cycle were all enhanced by flutamide administration during adrenarche in young female rats (Fig. 4A), thus active androgens (possibly testosterone) transformed from adrenal sex steroid precursors (e.g., DHEA) likely prevent NAFLD by suppressing glycolysis-mediated acyl-CoA biosynthesis and then de novo fatty acid biosynthesis in liver. Accordingly, we proposed a putative mechanism for adrenarche-accompanied androgens to prevent NAFLD in young female rats and shown in Fig. 10B. Inactivating SREBF1 signaling seems to be the key mechanism in mediating adrenarche-accompanied androgens to prevent hepatic de novo fatty acid biosynthesis, but through which mechanism it suppresses hepatic cell cycle progression is not clear (Fig. 10B).

## Discussion

NAFLD has rapidly emerged as the predominant contributor to chronic liver disease in children and adolescents, however its etiology is largely unknown [24]. In this study, we firstly reported that the adrenarche phase in young females is a period very susceptible for developing NAFLD and, the increase of adrenal-derived sex steroid precursors during this period is very critical in preventing the incidence of NAFLD in young females by suppressing hepatic de novo fatty acid biosynthesis and meanwhile enhancing fatty acid oxidation via converting into active androgen hormones. Any interruption of adrenal sex steroid precursor hormone production and/or their conversion into active androgens during adrenarche, thus, may cause NAFLD in young female children.

During adrenarche, the adrenal glands start to produce various sex steroids. Among them, dehydroepiandrosterone (DHEA) is the most abundant sex steroid precursors produced during this period [25]. Both in vivo and in vitro studies have strongly evidenced that adrenal-derived DHEA plays a great role in preventing hepatic lipogenesis and thus the development and incidence of NAFLD [14, 25, 26]. Thus, the increase of adrenal-derived DHEA during adrenarche may serve as an important factor to suppress hepatic lipogenesis and prevent the incidence of NAFLD in young females. DHEA itself is biologically inactive, it is only converted into active steroid hormones in the specific target tissues like the liver where the appropriate enzymatic machinery exists [25]. In the present studies, we found that specifically blocking androgen actions across adrenarche by administrating AR antagonist flutamide predominantly promoted hepatic de novo fatty acid synthesis and thus caused severe NAFLD in young female rats. In contrast, precisely preventing the conversation of adrenal sex steroid precursors into active estrogens by administrating aromatase inhibitor, formestane across adrenarche phase had minimal effects on hepatic de novo fatty acid synthesis and liver fat deposition in young female rats. Taken together, these results suggest that conversion into active androgens seems to be essential for adrenal sex steroid precursors to prevent hepatic de novo fatty acid synthesis and thus the incidence of NAFLD in young females during adrenarche. In support, we further evidenced that liver lipogenesis and fat deposition were markedly reduced once further enhancing androgen actions across adrenarche by administrating DHT, a nonaromatic androgen, in young female rats. Considering DHEA is the most abundant adrenal-produced androgen precursor [25], we proposed that DHEA is the most potential adrenal sex steroid precursor that exerts anti-NAFLD effect in young female rats by converting into active androgens.

However, in contrast to our findings that a previous study in adult female mice has confirmed that the conversion into estradiol rather than testosterone is a necessary prerequisite for DHEA to prevent the development and incidence of NAFLD [14]. And, they further verified that the DHEA-converted active estrogens exert its anti-NAFLD effect through precisely activating G protein-coupled estrogen receptor (GPER1, also called GRP30) rather than the nuclear estrogen receptors in liver [14]. Through RNA-seq, we found that *Cper1* did not express in liver of young female rats. This could well explain that why blocking the conversation of adrenal sex steroid precursors like DHEA into active estrogens by using aromatase inhibitor, formestane had minimal effects on hepatic de novo fatty acid synthesis and liver fat deposition in young female rats. Therefore, whether it is through converting into active androgens or into active estrogens for adrenal sex steroid precursors to exert their anti-NAFLD effect in females, seems to be age- or development-dependent. Based on the previous [14] and current studies, it is likely through converting into active androgens in young ages, but through converting into active estrogens in adult ages. If so, the precise mechanism triggering this transition between different age is intriguing and warrants deeper investigations. Another possibility is that such mechanism is species-dependent. As the previous studies that reported DHEA exerts anti-NAFLD effect through converting into active estrogens were performed in mice, while it is worth noting that our present studies were conducted in rats.

Active androgens (e.g., testosterone) exert their actions by binding to and activating the nuclear transcription factor AR [19]. To further understand the underlying mechanisms by which active androgens converted from adrenal sex steroid precursors prevent liver lipogenesis and in turn the incidence of NAFLD in young female rats, we searched for DEGs with potential binding sites for AR using CiiiDER software [20]. Resultantly, we found almost no DEGs involved in de novo fatty acid synthesis/cell cycle were with potential AR binding sites within their promoter regions, suggesting active androgens derived from adrenal androgen precursors exert anti-NAFLD effect through indirect pathways. Another important nuclear transcription factor, sterol regulatory element-binding transcription factor 1 (SREBF1) encoded by *Srebf1* was evidenced to be an important transcriptional target of AR [21]. Meanwhile, SREBF1 has been well established to serve as a gatekeeper of de novo lipogenesis in liver [22]. In the present study, *Srebf1* expression in liver was markedly upregulated by AR antagonist administration. Based on these results, there is a strong possibility that the anti-NAFLD effect of active androgens, converted from adrenal sex steroid precursors, is exerted through the indirect inactivation of hepatic SREBF1 signaling in young females. In support of our speculation, SREBF1 was evidenced to operate as a key metabolic effector of AR to orchestrate de novo lipid synthesis in cancer cells [21]. Using CiiiDER software, as expected, we found a large number of DEGs involved in lipid biosynthetic process are with potential SREBF1 binding sites within their promoter regions. Among them, the well-known SREBF1 target de novo fatty acid biosynthesis key genes, e.g., *Acly*, *Acaca*, *Fasn*, *Elovl6* and *Scd1* [22] were identified (Fig. 10A), and all of them were largely upregulated by AR antagonist administration. Accordingly, we proposed that SREBF1 should play an essential role in mediating adrenarche-accompanied androgens to prevent hepatic de novo lipogenesis and thus the incidence of NAFLD in young females.

Besides, a quantity of well-known fatty acid oxidation genes (e.g., *Ppara*, *Cpt2*, *Acox1*, etc.) were also identified to contain potential SREBF1 binding sites within their promoter regions. And, their expressions were all downregulated following AR antagonist administration across adrenarche in young female rats. Thus, it seems that, excepting potentiating de novo fatty acid synthesis, SREBF1 may be also able to inhibit fatty acid oxidation, thereby synergistically promoting fat deposition in liver. In support, SREBF1 has been evidenced to potently inhibit fatty acid oxidation in hepatocellular carcinoma [27]. Taken together, SREBF1 transcriptional networks likely play a central role in mediating active androgens converted from adrenal androgen precursors to prevent the incidence of NAFLD in young females by both inhibiting de novo fatty acid biosynthesis and meanwhile promoting fatty acid oxidation in liver.

Excepting preventing hepatic de novo fatty acid biosynthesis and the incidence of NAFLD, we also found that adrenarche-accompanied adrenal steroids exerted a predominant negative impact on liver growth. Transcriptomic profiling further verified that the hepatic cell cycle in young female rats are highly susceptible to active androgens during adrenarche as well. It is well established that fatty acids serve as important building blocks for the synthesis of cell membrane phospholipids, which in turn are essentially required for cells to divide and proliferate [28, 29]. Thus, the transient slowdown of liver growth during adrenarche in young female rats is likely due to blocked hepatic cell cycle progression as a result of the adrenal androgen-triggered decrease of hepatic de novo fatty acid synthesis. Accordingly, it appears that the slowdown of liver growth in early life could be applied as an indicator of the onset of adrenarche in young females.

Mechanistically, using CiiiDER we identified *Cdca3* (encoding cell division cycle-associated protein 3, CDCA3) with putative SREBF1 binding sites within its promoter region. CDCA3 participates in forming E3 ligase complex with S-phase kinase-associated protein 1 (SKP1) and cullin 1 (CUL1), which is required for cell mitosis entry [30]. And it has been widely evidenced in multiple human cancers that silencing *Cdca3* would cause cell cycle arrest and block cell proliferation [30, 31]. Therefore, *Cdca3* may exert an important role in mediating SREBF1 to block hepatic cell cycle progression in young females during adrenarche. Even though, among the totally 43 DHT-downregulated and cell cycle involved DEGs (Fig. 5C), excepting *Cdca3*, no other DEGs were identified to contain putative SREBF1 binding sites within their promoters. Thus, it seems that SREBF1 is not the primary factor, or at least not acts in a direct manner, to mediate adrenarche-accompanied androgens to suppress hepatic cell proliferation in young female rats. Therefore, further investigations are still needed to identify the key genes that mediate active androgens to block liver cell cycle progression and its growth during adrenarche in young females.

Paradoxically, in our studies, further enhancing androgen actions by administrating DHT across adrenarche in young female rats notably reduced liver TG content, but without effects on liver *Srebf1* expression as well as the expression of its target genes involved in either de novo fatty biosynthesis or fatty acid oxidation. This was presumably due to the timing of sampling being too late to see the desired results. Because, previous studies have revealed that essential fatty acids deficiency would promote lipogenic gene expression in liver [32]. Thus, initially DHT administration may indeed decrease the expression of these key lipogenic genes in liver. But, with time ongoing, essential fatty acids deficiency occurred which in turn caused upregulation of these lipogeneic genes, thereby interacting the regulatory action of DHT.

Based on our results, it seems in females, the adrenal-derived sex steroid hormones play crucial roles in preventing NAFLD in childhood, and then transmitting to ovary-secreted estrogens [33] from puberty to menopause. After menopause, it has reported that, with the decline of both ovarian estrogens [33, 34] and adrenal sex steroid precursors like DHEA [35], the incidence of NAFLD in women would be significantly increased. These results highlight the importance of sex steroid hormones secreted from both the adrenal glands and ovaries in protecting liver healthy in the life span of females. Nowadays, estrogen therapy is the mainstay clinical treat for NAFLD, especially in menopausal women [36], however it will inevitably causes endocrine disorders and increases the risk for breast cancer [37]. In contrast, adrenal-derived sex steroid precursors like DHEA are only converted into active steroid hormones within the specific target tissues [38], thus largely limiting their adverse effects compared to exogenous estradiol. Thus adrenal-derived sex steroid precursors like DHEA [14] could be developed as new promising chemicals or drugs for the prevention and treatment of NAFLD in young female children.

## Conclusion

Collectively, we firstly evidenced that adrenarche-accompanied rise of adrenal-derived sex steroid precursors is associated with rewiring of hepatic metabolic pathways, including suppressing de novo fatty acid synthesis and blocking cell cycle in liver by converting into active androgens in young females. SREBF1 transcriptional networks appears to play a central role in mediating active androgens converted from adrenal-derived sex steroid precursors to prevent hepatic de novo lipogenesis and the incidence of NAFLD in young females. Our novel finding provides new insights into the etiology of NAFLD and is crucial in developing effective prevention and management strategies for NAFLD in female children.

## Limitations

The results of the present study provide novel insights into the etiology of NAFLD in young females. However, there are still several limitations in this study. Firstly, which adrenal sex steroid precursor(s) exerts the key role in preventing the incidence of NAFLD in young females is unknown and requires further investigations. Secondly, although through integrative analysis we identified that AR-SREBF1 transcriptional networks appears to play a central role in mediating adrenarche-accompanied adrenal androgens to prevent the incidence of NAFLD in young females, it still requires further validation using like liver-specific Ar/*Srebf1* knockout animal models. Thirdly, the mechanisms by which adrenal sex steroid precursors slowdown liver growth during adrenarche in young females is unknown and require further investigation. Fourthly, our studies clearly evidenced that the conversion into active androgens rather than estrogens is a necessary prerequisite for adrenal sex steroid precursors to prevent the incidence of NAFLD in young female rats, which is completely opposite to a previous report in adult mice [14]. The reasons and mechanisms contributing to this different conclusion between studies are interesting and remain to be elucidated. Especially, further exploring the hepatic AR signaling pattern and its association with hepatic lipid metabolism during adrenarche may facilitate to clarify such paradox. Finally, our conclusion was drawn based on studies conducted in young female rats, whether the same is true for female children is unknown and requires further validation.

## Materials and methods

### Animals and treatment

Male and female Sprague–Dawley (SD) rats at age of 10–12 wk of age, purchased from the HuaXi Laboratory Animal Center of Sichuan University, were bred to generate pups using for this study. Rats were given *ad libitum* access to a commercial diet and tap water in a controlled environment with temperature of 21 ± 1 °C, a relative humidity of 50–60% and a 12 h light/12 h dark cycle.

### Experiment 1: female rat liver growth profile

To profile female rat liver growth from neonatal to adult age, 8 female rats were weighted and decapitated at age of 1, 2, 3, 4, 5, 6 and 8 wk of age, respectively. Before decapitation, the blood samples were collected and, centrifuged at 2000×g for 15 min at 4 °C and sera were stored at -20 °C pending analysis of hormone concentrations. After decapitation, the liver was isolated immediately and weighted. And the liver index was calculated by dividing its weight by total body weight. Then the liver samples were frozen in liquid nitrogen and then stored at -80 °C pending further use.

### Experiment 2: Effects of adrenal sex steroid precursors-converted androgens on liver growth and liver fat deposition during adrenarche in young female rats

For investigating the effects of adrenal-derived androgens on liver growth in early life, 36 female pups at 2 wk of age with similar body weight were selected from 8 different litters and randomly allocated to one of three groups. Briefly, 13 rats were i.p., administrated with flutamide (Sigma–Aldrich, St.Louis, MO, USA; suspended in corn oil, CAS# 13311-84-7) at a dose 50 mg/kg [18], 12 rats were i.p., administrated with DHT (dihydrotestosterone, Sigma–Aldrich, St.Louis, MO, USA; CAS# 521-18-6; suspended in corn oil) at a dose of 5 mg/kg, and 11 rats were given placebo injections (corn oil) and served as controls (Vehicle). Injections were performed every two days at 0800 AM for 2 weeks, and body weight of each individual rat was weighted before injection. All the female pup rats were housed with their mothers until sacrifice at the end of the experiment (i.e., 4 wk of age).

At 4 wk of age, all female pup rats were anaesthetized with ether and then decapitated. The blood samples (1-1.5 mL) were collected, centrifuged at 2000 × g for 15 min at 4 °C and sera were stored at -20 °C pending analysis of hormone and lipid concentrations. After decapitation, the liver was immediately isolated and weighed. Three portions of liver samples were collected at the same lobule. For RNA-seq, liver samples from 3 ~ 4 individual rats were pooled together as a single biological replicate, and three biological replicates in each group were used. The remaining one portion from each rat was fixed in Bouin’s solution for histology analysis, and the last portion was frozen in liquid nitrogen and then stored at -80 °C for gene expression analysis and oil red O staining analysis .

### Experiment 3: Effects of adrenal sex steroid precursors-converted estrogens on liver growth and liver fat deposition during adrenarche in young female rats

For exploring the effects of adrenal sex steroid precursors-converted estrogens on liver growth and liver fat deposition, 20 female pup rats at 2 wk of age with similar body weight were selected and allocated into one of two groups. Briefly, 10 rats were i.p., administrated with formaestane (4-hydroxyandrost-4-ene-3,17-dione; Sigma–Aldrich, St.Louis, MO, USA; CAS# 566-48-3; suspended in corn oil) at a dose of 17.5 mg/kg body weight, as suggested previously [39]. And, the remaining 10 rats were i.p., administrated with corn oil (Vehicle). Injections were performed every two days at 0800 AM for 2 weeks, and body weight of each individual rat was weighted before injection. All the female pup rats were housed with their mothers until sacrifice at the end of the experiment (i.e., 4 wk of age). After decapitation, the liver was immediately isolated and weighed. Then the same liver lobule of each individual rats was frozen in liquid nitrogen and then stored at -80 °C for gene expression and Oil Red O staining analyses.

### RNA sequencing and data analysis

For RNA-seq, liver samples from 3 ~ 4 individual rats were pooled together as a single biological replicate, and three biological replicates in each group were used. RNA sequencing and data analysis were performed as our previous descriptions [40]. Namely, RNA was extracted from the pooled liver samples using TRIzol (Invitrogen, Carlsbad, CA, USA). The quality of the total RNA was checked using the Agilent 2100 Bioanalyzer system (Santa Clara, CA, USA). A total amount of 1 µg RNA per sample with RNA integrity numbers (RINs) of 8.5 or greater was used as input material for the RNA sample preparations. Sequencing libraries were generated using NEBNext® UltraTM RNA Library Prep Kit for Illumina® (NEB, USA) following manufacturer’s recommendations. Briefly, mRNA was extracted from total RNA using oligo (dT) magnetic beads and sheared into short fragments of about 200 bases. These fragmented mRNAs were then used as templates for cDNA synthesis. The cDNAs were then PCR amplified to complete the library. The cDNA library was sequenced on a 50-cycle single end run in HiSeq 2500 (Illumina) by Novogene Co., Ltd (Beijing, China). Raw RNA-Seq reads were processed through in-house perl scripts. Clean reads were obtained by removing reads containing low quality reads, adaptor sequences and reads containing ploy-N from raw reads, and mapped to the rat genome (Rnor 6.0) using Hisat2 software. The gene expression level was then calculated using the reads per kilo bases per million reads (RPKM) method. Pairwise comparisons between groups were performed using the DESeq2 R package (1.16.1). The resulting *P*-values were adjusted using the Benjamini and Hochberg’s approach for controlling the false discovery rate. Genes with an adjusted *P* value (q value) < 0.05 were assigned as differentially expressed (DEGs) [41]. Hierarchical cluster analysis was conducted to assemble genes with similar expression patterns across groups using Cluster 3.0 software. After calculation of the z-score for each gene, gene clustering was performed with an average linkage method with the Euclidean distance.

### Functional enrichment analysis

For Gene Ontology (GO) and Kyoto Encyclopedia of Genes and Genomes (KEGG) pathway enrichment analyses of DEGs, the Database for Annotation, Visualization and Integrated Discovery (DAVID) Bioinformatics Resources (v2022q4) was used. In all tests, *P* values were calculated using the Benjamini-corrected modified Fisher’s exact test and *P* < 0.05 was taken as a threshold of significance. The DEGs’ functional enrichment network figures were made using Gephi software (v0.10.1).

Gene Set Enrichment Analysis (GSEA) was performed using GSEA (v4.3.2) software. Genes including all DEGs and non-DEGs were pre-ranked based on the -log10 (*p*-value) multiplied by the sign of gene log2 Fold Change such that the up-regulated genes had positive scores and down-regulated had negative scores. This application scores a sorted list of genes with respect to their enrichment of selected functional categories(Gene Ontology [GO], Kyoto Encyclopedia of Genes and Genomes[KEGG] and Reactome). Terms annotating more than 500 or less than 5 genes were discarded. The significance of the enrichment score was assessed using 1000 permutations and default of *P* value < 0.05 was considered significant.

### Mapping of AR/SREBF1 binding sites

The potential AR/SREBF1 binding sites across the promoter region (1500 bp upstream and 500 bp downstream of the transcription start site) of the core genes were scanned by CiiiDER software. JASPAR2020 CORE_vertebrates clustering was used as the transcription factor position frequency matrix.

### Serum hormones assays

Serum DHEA concentrations were quantified by commercial RIA Kits (Enzo Life Sciences, Farmingdale, NY, United States). The sensitivity of the assay was 2.9 pg/ml ng/mL, with intra- and inter-assay CVs of 5.66 and 7.9%, respectively. The DHEA assay had a cross-reactivity of less than 1% for progesterone (0.06%), testosterone (0.1%), androstenedione (0.73%), and androsterone (0.29%).

### Serum and liver lipid level assays

Triglyceride (TG) (Cat#A110-1-1) and free fatty acid (Cat#A042-2-1) levels in both serum and liver were detected by the commercial kits (Nanjing Jiancheng Biotechnology Institution, China) following the manufacturer’s instructions.

### Histology analysis

The liver from each rat was collected and fixed in 4% paraformaldehyde for 48 h, and then paraffin embedded. Then, the liver was serially sectioned at 5-µm thickness, and were subjected to standard H&E staining for morphological observation, as described previously [14].

### Oil Red O staining analysis

For lipid deposition evaluation, 10 μm thick sections of frozen liver tissues were made and stained with Oil Red O (Cat#D027-1; Nanjing Jiancheng Biotechnology Institution, China) following the manufacturer’s instructions.

### Relative real-time quantitative PCR (qPCR)

Total RNA was isolated from the liver according to the manufacturer’s instructions (Invitrogen Co., Carlsbad, CA, USA). Quantitative and qualitative analyses of isolated RNA were assessed from the ratio of absorbance at 260 and 280 nm and agarose gel electrophoresis. A total of 1000 ng RNA was converted into first-strand cDNA using a PrimeScript® RT reagent kit with a gDNA Eraser (TaKaRa Bio, Co., LTD, Dalian, China). The RT-PCR was undertaken on a CFX96 Real-Time PCR detection system (BioRad, Hercules, California, USA). The PCR reaction contained 1 µL cDNA, 500 nmol/L each of forward and reverse primers, and 2×SYBR® premix TaqTM (TaKaRa Bio Co., Ltd., Dalian, China). Primer sequences of target and reference genes are shown (Supplemental file 4). The PCR cycling conditions were as follows: initial denaturation at 95 °C (1 min), followed by 40 cycles of denaturation at 95 °C (5 s), annealing at 60 °C (25 s) and a final melting curve analysis (to monitor the PCR product purity). A reference housekeeping gene (*Gapdh*) was measured for each sample. The amplification efficiency of each RT-PCR primer, measured using a standard curve method, was within 90–110%. The fold change of mRNA in the treatment group relative to the control group was determined using 2^−ΔΔCt^.

### Statistical analyses

Statistical analysis was performed using GraphPad Prism 9.2 (La Jolla, CA) software. Comparisons among groups were carried out via one-way ANOVA followed by Turkey’s test. For the analysis of the effect of treatment in repeated measures (i.e., body weight and serum DHEA), two-way ANOVA followed by Sidak’s multiple comparisons test was used. All values were expressed as the mean ± SEM and statistical significance was defined as *p* < 0.05.

### Supplementary Information

**Supplementary Material 1.**

**Supplementary Material 2.**

**Supplementary Material 3.**

**Supplementary Material 4.**

## Data Availability

All data generated or analyzed during this study are included in this published article and its Supplementary files. The RNA sequencing raw data generated during the current study is available in the GSA database (https://bigd.big.ac.cn/gsa/browse; Accession: CRA011013).

## Abbreviations

NAFLD: Non-alcoholic fatty liver disease
DHEA: Dehydroepiandrosterone
Srebf1: Sterol regulatory element-binding transcription factor 1
DHT: Dihydrotestosterone
FMT: Formestane
FPKM: Fragments per kilobase of transcripts per million
GO: Gene ontology
KEGG: Kyoto Encyclopedia of Genes and Genomes
BP: Biological process
MF: Molecular Function
CC: Cellular Component
GSEA: Gene Set Enrichment Analysis
