# Supplementary material for: Adrenarche-accompanied rise of adrenal sex steroid precursors prevents NAFLD in Young Female rats by converting into active androgens and inactivating hepatic Srebf1 signaling
Source: BMC Genomics. 2024 Feb 19;25:190. doi: 10.1186/s12864-024-10107-6 (PMC10875776; doi:10.1186/s12864-024-10107-6)
Supplement: Supplementary file 3 — Supplementary Material 3. [file 12864_2024_10107_MOESM3_ESM.doc]

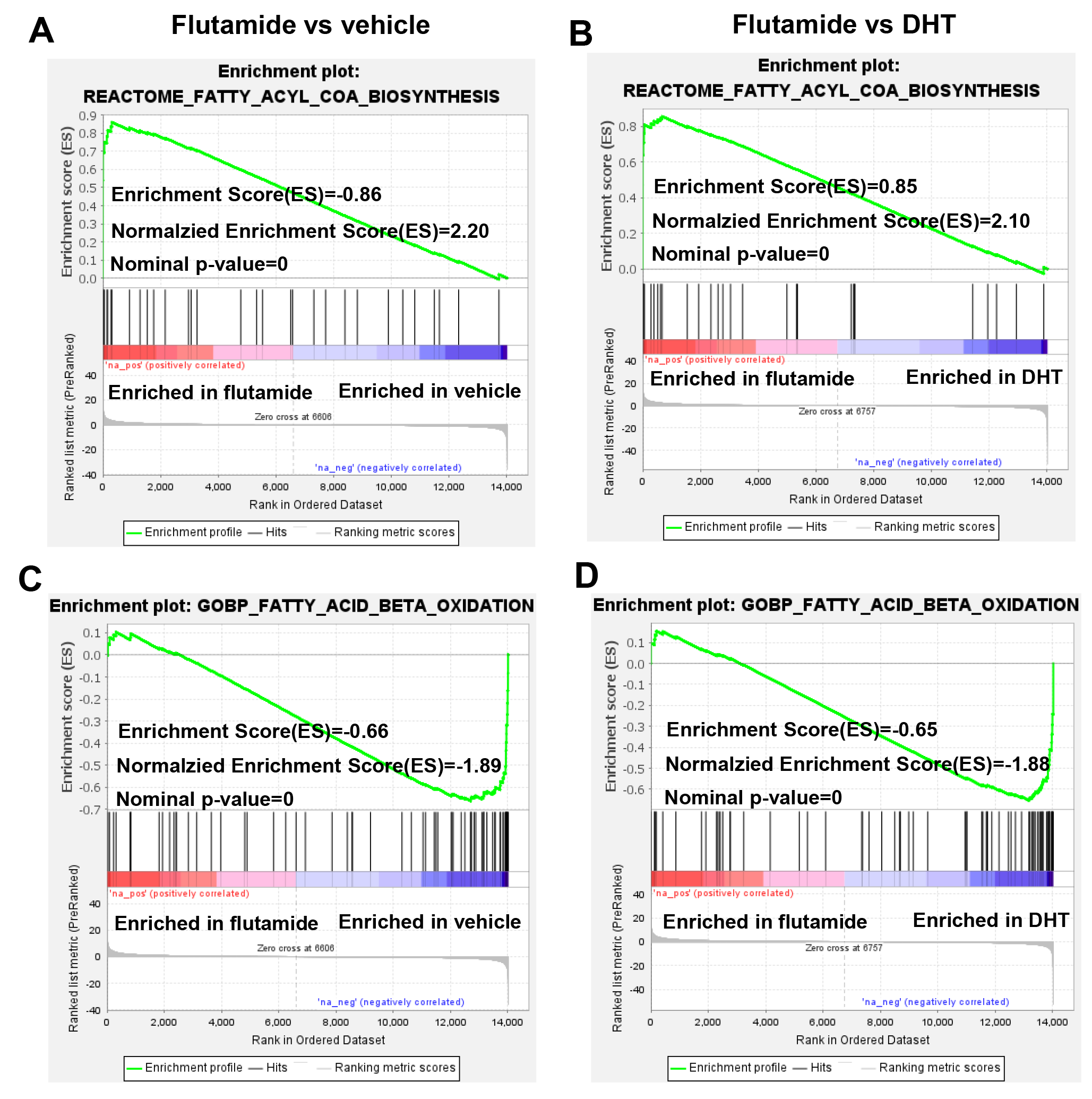


**Figure S1.** Gene set enrichment analysis (GSEA) indicated that flutamide administration across adrenarche promoted *de novo* fatty acid synthesis and suppressed fatty acid oxidation. (**A-B**) GSEA indicated that flutamide administration across adrenarche promoted hepatic ATP citrate lyase (ACLY)-mediated fatty acid synthesis in young female rats. (**C-D**) GSEA shown that flutamide administration across adrenarche suppressed hepatic fatty acid beta oxidation in young female rats.
