# Supplementary material for: Adrenarche-accompanied rise of adrenal sex steroid precursors prevents NAFLD in Young Female rats by converting into active androgens and inactivating hepatic Srebf1 signaling
Source: BMC Genomics. 2024 Feb 19;25:190. doi: 10.1186/s12864-024-10107-6 (PMC10875776; doi:10.1186/s12864-024-10107-6)
Supplement: Supplementary file 4 — Supplementary Material 4. [file 12864_2024_10107_MOESM4_ESM.doc]

qPCR Primer sequences for tissue genes

| **Gene** | **Genbank accession no.** | **Primer sequence (5’-3’)** | **Amplification length (bp)** |
| --- | --- | --- | --- |
| Srebf1 | NM_001276707.1 | F: TCACAGATCCAGCAGGTCCCC  R: GGTCCCTCCACTCACCAGGGT | 180 |
| Acly | NM_016987.2 | F: GGGAGAAGTTGGGAAGACCA  R: GTGCTCCCACTGGCATTAAG | 162 |
| Acaca | NM_022193.2 | F: GAGGTGGATCAGAGATTTCA  R: TTCAGCTCTAACTGGAAAGC | 125 |
| Fasn | NM_017332.2 | F: AGGATGTCAACAAGCCCAAG  R: ACAGAGGAGAAGGCCACAAA | 100 |
| Elovl6 | NM_134383.3 | F: AGATGCTGATGGGCTGTCTC  R: TGAGTGAGGACCAGAAGATG | 105 |
| Scd1 | NM_139192.2 | F: CAGAGCCAGGTGCCACTTTT  R: TGCTAGAGGGTGTACCAAGCTTT | 104 |
| Cpt1a | NM_031559.2 | F: GGATGGCATGTGGGTAAAAG  R: TACTGACACAGGCAGCCAAA | 204 |
| Ppara | NM_013196.2 | F: AATCCACGAAGCCTACCTGA  R: GTCTTCTCAGCCATGCACAA | 132 |
| *Gapdh* | NM_017008.4 | F: CGGCAAGTTCAACGGCACAG  R: ACTCCACGACATACTCAGCAC | 134 |

*Abbreviations: Srebf1*, sterol regulatory element binding transcription factor 1; *Acly*, ATP citrate lyase; *Acaca*, acetyl-CoA carboxylase alpha; *Fasn*, fatty acid synthase; *Elovl6*, ELOVL fatty acid elongase 6; *Scd1*, stearoyl-CoA desaturase 1; *Cpt1a*, carnitine palmitoyltransferase 1A; *Ppara*, peroxisome proliferator activated receptor alpha; *Gapdh*, glyceraldehyde-3-phosphate dehydrogenase.
